# Outcomes of Cardiopulmonary Resuscitation and Predictors of Its Outcomes in the Emergency Department in King Saud Medical City, Saudi Arabia

**DOI:** 10.7759/cureus.39268

**Published:** 2023-05-20

**Authors:** Mustafa Alhaj Zeen, Joud Aburisheh, Saleh S Alshehri, Shouq A Alshehri, Fatema S Smaisem, Huda Hijazi, Mohammed M Alamri, Asmaa Hegazy

**Affiliations:** 1 Faculty of Medicine, Almaarefa University, Riyadh, SAU; 2 Pediatric Intensive Care Unit, King Saud Medical City, Riyadh, SAU; 3 Internal Medicine Department, King Saud Medical City, Riyadh, SAU

**Keywords:** survival-to-discharge, return of spontaneous circulation (rosc), emergency department cpr, cardiopulmonary resuscitation knowledge, in hospital cardiac arrest

## Abstract

Background: Cardiac arrest is a medical emergency marked by the cessation of cardiac mechanical activity and insufficient blood flow. CPR (cardiopulmonary resuscitation) is a life-saving method that involves restoring the essential functions of two vital organs: the heart and lungs. This study was conducted to identify the outcome of CPR in cardiac arrest patients presented to the emergency department (ED) and to identify predictors of CPR outcomes.

Methodology: This was a retrospective, descriptive study. All in-hospital cardiac arrest patients who underwent CPR in the King Saud Medical City (KSMC) ED between January 2017 and January 2020 were analyzed, with a sample size of 351 patients.

Results: Overall return of spontaneous circulation (ROSC) and survival to discharge (STD) were achieved in 106 (30.2%) and 40 (11.39%) patients, respectively. When assessing the predictors of ROSC, the analyses showed that patient age, pre-arrest intubation, the method used to deliver oxygen, and CPR duration were all statistically significant predictors for ROSC. Similarly, when assessing predictors associated with STD, the analyses showed that patient age, pre-arrest intubation, the method used to deliver oxygen, and CPR duration were positively associated with STD.

Conclusion: Comparing the study's findings to those of similar studies, it shows a CPR outcome rate within the range of similar studies. It also highlights that CPR outcomes are highly associated with CPR duration (a maximum of 30 minutes), younger age, and endotracheal intubation.

## Introduction

Cardiac arrest is a medical emergency marked by the cessation of cardiac mechanical activity and insufficient blood flow, as evidenced by the lack of a palpable central pulse, apnea, loss of pulse, blood pressure, and spontaneous breathing [1]. Although the problem may be reversible with appropriate care, if proper action is not taken, it can result in death [1]. Heart disease, trauma, pulmonary dysfunction, poisoning, malignancies, a variety of strokes, electrocution, drowning, frostbite, and other medical conditions can all cause cardiac arrest [2].

CPR (cardiopulmonary resuscitation) is a life-saving method that involves restoring the essential functions of two vital organs: the heart and lungs [3,4]. In a person suffering cardiac arrest, CPR is an attempt to restore spontaneous blood circulation and breathing [3,4]. Return of spontaneous circulation (ROSC) is achieved if the procedure is completed successfully. The term "ROSC" refers to the restoration of a pulse and its maintenance for more than 20 minutes [5]. One more important CPR outcome is survival to discharge (STD), which is defined as a patient being transferred from the ICU to the telemetry unit, transferred from one facility to a lower level of care facility, or transferred to a nursing facility in a stable condition [5]. Unfortunately, CPR's effectiveness rate has remained consistent for the past 30 years [6]. An additional sobering fact is that in many circumstances (either due to technical deficiencies or an untrained workforce), the normal CPR protocol is not followed [7].

The emergency department (ED) of a hospital is responsible for most cardiac arrest patients, whether they are brought in from outside the hospital or are critically ill patients who go into cardiac arrest while receiving initial care in the ED. Existing research shows that a variety of factors influence the outcome of CPR done in the emergency room. Patient variables such as age and gender are included, as well as clinical and CPR-specific characteristics such as cardiac arrest mechanism, initial rhythm observed after cardiac arrest, clinical environment, response time, and CPR duration, among others [8-10].

It is becoming more vital to investigate pre-arrest and arrest parameters and gain better knowledge of their prognostic implications so that high-quality CPR may be given in a rational, productive, and successful manner to those patients who would benefit the most. This study was conducted to identify the outcomes of CPR in cardiac arrest patients presented to the emergency department at King Saud Medical City in Riyadh, Saudi Arabia, and identify predictors associated with improved outcomes.

## Materials and methods

Subjects and study design

This was a retrospective, descriptive study of all in-hospital cardiac arrest patients who underwent CPR in the King Saud Medical City (KSMC) ED for a period of three years, from January 2017 to January 2020. KSMC is a tertiary care center located in Riyadh, the capital city of Saudi Arabia.

Inclusion

Patients who had sustained an in-hospital cardiac arrest in the ED and underwent CPR between January 2017 and January 2020 were included. The medical records of 351 patients were included in this study's final analysis.

Exclusion

Out-of-hospital cardiac arrest patients (age < 14 years old), those who were reported dead on hospital arrival, and those with missing records or incomplete data were excluded. Those with existing do-not-resuscitate orders and those who were immediately shifted to another facility for post-arrest care were excluded from the final analysis. Additionally, in patients who had more than one episode of cardiac arrest, the first event was considered to be the seminal event.

Data collection

Data were extracted from the KSMC emergency database, electronic medical records, and patient paper notes on patient demographics, clinical history, and cardiac arrest clinical features such as location, likely cause of arrest, monitored arrest, and initial rhythm. CPR variables such as the time that CPR started, CPR duration, and intubation during CPR were included in the study. The CPR outcomes were determined to be ROSC and STD for the purpose of this research. Patients who were discharged home from the hospital as well as those who were moved to another facility after a minimum of 24 hours following ROSC were included in the STD category.

Data analysis

Data were analyzed using SPSS version 26 (IBM Corp., Armonk, NY) and Microsoft Excel (Microsoft® Corp., Redmond, WA) to generate tables and charts with a P-value of less than 0.05 considered significant. Means and standard deviation were recorded for continuous variables for descriptive analysis. As previously mentioned, ROSC and STD were the outcomes of interest.

Ethical consideration

Ethical approval from the Institutional Review Board (IRB) of King Saud Medical City (IRB registration number: H-01-R-053) was obtained before data collection began. Regarding confidentiality and privacy, the personal information (name and contact information) of patients was not included in the study. Since we used medical records, informed consent was waived.

## Results

Three hundred and fifty-one patients presented to the ER-KSMC. In total, 264 (75.2%) were males, and 87 (24.8%) were females. More than 60% were of cardiac etiology (212 patients); 45 patients (12.8) had respiratory etiologies; all other etiologies were less than 10%. As for the pre-arrest characteristics of these patients, 12 (3.4%) were on dialysis, 147 (41.9%) were intubated, 304 (86.6%) had IV access, 95 (55.6%) were under mechanical ventilation, 345 (98.3%) were monitored, 169 (48.1%) were receiving IV vasopressors, 164 (46.7%) of patients’ blood pressures were not recorded, 162 (46.2%) were hypotensive, 17 (4.8%) were hypertensive, and 8 (2.3%) were found to have normal blood pressure. More than 328 patients (93%) received the first dose of epinephrine within less than five minutes, and only 23 (6.6%) patients received the first dose of epinephrine after more than five minutes. Results showed that an endotracheal tube was used on 128 of the patients (36.5%). It was placed at the time of the event, and they were not reintubated again after the cardiac arrest took place. About 19 of the patients (5.4%) were reintubated, and 130 (37%) were intubated during the event of CPR. Almost 74 of the patients (21.1%) required an ambu bag instead of an endotracheal tube. Regarding the rhythm, results showed that 173 (49%) of the patients had an asystole rhythm, 120 (34.2%) had a rhythm of pulseless electrical activity, and 35 (10%) were found to have bradycardia. As for shockable rhythms, 16 (4.6%) of the patients had ventricular fibrillation, and 7 (2%) had ventricular tachycardia (Table 1).

**Table 1:** Characteristics of all patients. IV: intravenous, NR: no record, PEA: pulseless electrical activity, VF: ventricular fibrillation, VT: ventricular tachycardia.

		Total% (n=351)
Gender	Male	264 (75.2%)
	Female	87 (24.8%)
Etiology	Cardiac	212 (60.4%)
	Non-cardiac	33 (9.4%)
	Respiratory	45 (12.8%)
	Trauma	17 (4.8%)
	Unknown	44 (12.5%)
Blood press	NR	164 `(46.7%)
	Hypotension	162 (46.2%)
	Normal	8 (2.3%)
	Hypertension	17 (4.8%)
On dialysis		12 (3.4%)
Intubated		147 (41.9%)
IV access		304 (86.6%)
Mechanical ventilation		195 (55.6%)
Intravenous vasopressor		169 (48.1%)
Rhythm	Asystole	173 (49.%)
	PEA	120 (34.2%)
	Bradycardia	35 (10%)
	VF	16 (4.6%)
	VT	7 (2%)
Monitored		345 (98.3%)
First dose epinephrine	First dose epinephrine <5 min n(%)	328 (93.4%)
	First dose epinephrine >5 min n(%)	23 (6.6%)
Method used to deliver oxygen	Endotracheal tube in place at time of event	128 (36.5%)
	Endotracheal tube placement during event	130 (37%)
	No endotracheal tube usage	74 (21.1%)
	Reintubated	19 (5.4%)

**Figure 1:**
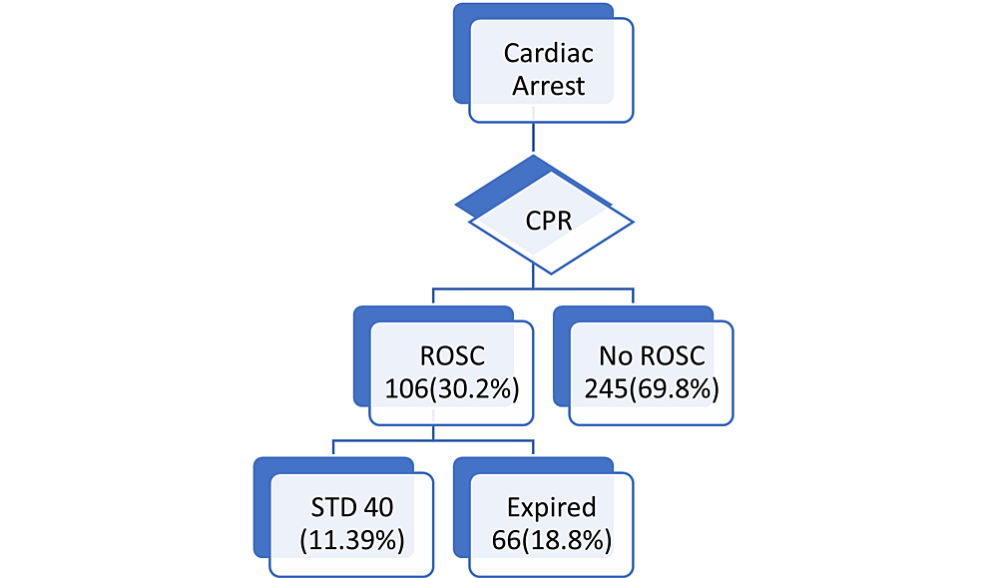
Outcome of cardiopulmonary resuscitation in cardiac arrest patients. ROSC: return of spontaneous circulation, STD: survival to discharge.

Figure 1 shows the CPR outcome of patients who had cardiac arrests in the KSMC ED. ROSC was achieved in 30.2% of patients, while 11.39% were discharged alive.

The mean age for all patients was 56.6 years (±14.7 SD). The ROSC group had an average age of 52.17 (±13.98 SD), compared with the STD group, which had an average age of 48.3 years (±15.7 SD). The mean CPR duration for all patients was 26.5 minutes (±8 SD). The CPR duration was 23.4 minutes (±8.5 SD) for the ROSC group compared with the STD group, with an average duration of 18.3 minutes (±7.76 SD).

Males made up a larger fraction of the patients who achieved ROSC and STD (69.8% and 70%, respectively). Younger patients, compared to older patients, had a considerably higher chance of maintaining ROSC (p = 0.032), and the same was true for the STD group (p = 0.001). Analysis showed that as the CPR duration increases, the chance of maintaining ROSC (p=0.016) decreases, and the same is true for the STD group (p=0.015).

When assessing the predictors of ROSC, the analysis shows that patient age, pre-arrest intubated patient, method used to deliver oxygen, and CPR duration were all statistically significant for ROSC (Table 2).

**Table 2:** Assessing the predictors associated with return of spontaneous circulation. IV: intravenous, NR: no record, PEA: pulseless electrical activity, ROSC: return of spontaneous circulation, VF: ventricular fibrillation, VT: ventricular tachycardia.

Variable	ROSC	No	Yes	P-value
Gender	Male	72% (n=190)	28% (n=74)	0.123
	Female	63.2% (n=55)	36.8% (n=32)	
Age	Standard deviation 13.98 Mean 52.17			0.032
Etiology	Cardiac	70.8% (n=150)	29.2% (n=62)	0.135
	Non cardiac	66.7% (n=22)	33.3% (n=11)	
	Respiratory	55.6% (n=25)	44.4% (n=20)	
	Trauma	82.4% (n=14)	17.6% (n=3)	
	Unknown	77.3% (n=34)	22.7% (n=10)	
Blood pressure	NR	75% (n=123)	25% (n=41)	0.148
	Hypotension	66.7% (n=108)	33.3% (n=54)	
	Normal	62.5% (n=5)	37.5% (n=3)	
	Hypertension	52.9% (n=9)	47.1% (n=8)	
Dialysis	No	69.9% (n=237)	30.1% (n=102)	0.810
	Yes	66.7% (n=8)	33.3% (n=4)	
Intubated	No	63.7% (n=130)	36.3% (n=74)	0.003
	Yes	78.2% (n=115)	21.8% (n=32)	
IV access	No	66% (n=31)	34% (n=16)	0.537
	Yes	70.4% (n=214)	29.6% (n=90)	
Mechanical ventilation	No	70.5% (n=110)	29.5% (n=46)	0.795
	Yes	69.2% (n=135)	30.8% (n=60)	
IV vasopressor	No	58.1% (n=124)	31.9% (n=58)	0.480
	Yes	71.6% (n=121)	28.4% (n=48)	
Rhythm	Asystole	74.6% (n=129)	25.4% (n=44)	0.239
	PEA	68.3% (n=82)	31.7% (n=38)	
	Bradycardia	60% (n=21)	40% (n=14)	
	VF	56.3% (n=9)	43.8% (n=7)	
	VT	57.1% (n=4)	42.9% (n=3)	
CPR duration	Standard deviation 8.5 mean 23.4			0.016
Monitored	No	66.7% (n=4)	33.3% (n=2)	0.866
	Yes	69.9% (n=241)	30.1% (n=104)	
First dose epinephrine	Less than 5 min	70.7% (n=232)	29.3% (n=96)	0.151
	More than 5 min	56.5% (n=13)	43.5% (n=10)	
Method used to deliver oxygen	Endotracheal tube in place at time of event	78.9% (n=101)	21.1% (n=27)	<0.001
	Endotracheal tube placement during event	48.5% (n=63)	51.5% (n=67)	
	No endotracheal tube usage	90.5% (n=67)	9.5% (n=7)	
	Reintubated	73.7% (n=14)	26.3% (n=5)	

Similarly, when assessing predictors associated with STD, the analysis shows that patient age, pre-arrest intubated patient, the method used to deliver oxygen, and CPR duration were all associated with STD (Table 3).

**Table 3:** Assessing the predictors associated with survival to discharge. IV: intravenous, NR: no record, PEA: pulseless electrical activity, STD: survival to discharge, VF: ventricular fibrillation, VT: ventricular tachycardia.

Variable	STD	No	Yes	P-value
Gender	Male	89.4% (n=236)	10.6% (n=28)	0.471
	Female	86.2% (n=75)	13.8% (n=12)	
Age	Standard deviation 15.7 mean 48.3			0.001
Etiology	Cardiac	89.2% (n=189)	10.8% (n=23)	0.683
	Non-cardiac	90.9% (n=30)	9.1% (n=3)	
	Respiratory	82.2% (n=37)	17.8% (n=8)	
	Trauma	88.2% (n=15)	11.8% (n=2)	
	Unknown	90.9% (n=40)	9.1% (n=4)	
Blood pressure	NR	92.1% (n=151)	7.9% (n=13)	0.197
	Hypotension	85.8% (n=139)	14.2% (n=23)	
	Normal	75% (n=6)	25% (n=2)	
	Hypertension	88.2% (n=15)	11.8% (n=2)	
Dialysis	No	89.1% (n=302)	10.9% (n=37)	0.131
	Yes	88.5% (n=269)	11.5% (n=35)	
Intubated	No	84.3% (n=172)	15.7% (n=32)	0.003
	Yes	94.6%(n=139)	5.4%(n=8)	
IV access	No	89.4%(n=42)	10.6% (n=5)	0.861
	Yes	75% (n=9)	25% (n=3)	
Mechanical ventilation	No	87.2%(n=136)	12.8%(n=20)	0.453
	Yes	89.7%(n=175)	10.3%(n=20)	
IV vasopressor	No	87.4% (n=159)	12.6% (n=23)	0.448
	Yes	89.9% (n=152)	10.1% (n=17)	
Rhythm	Asystole	90.2% (n=156)	9.8% (n=17)	0.076
	PEA	90.8% (n=109)	9.2% (n=11)	
	Bradycardia	82.9% (n=29)	17.1% (n=6)	
	VF	68.8% (n=11)	31.3% (n=5)	
	VT	85.7% (n=6)	14.3% (n=1)	
CPR duration	Standard deviation 7.76 mean 18.3			0.015
Monitored	No	66.7% (n=4)	33.3% (n=2)	0.088
	Yes	89.0% (n=307)	11% (n=38)	
	Yes	87.5% (n=232)	12.5% (n=33)	
First dose epinephrine	Less than 5 min	88.4% (n=290)	11.6% (n=38)	0.673
	More than 5 min	91.3% (n=21)	8.7% (n=2)	
Method used to deliver oxygen	Endotracheal tube in place at time of event	95.3% (n=122)	4.7% (n=6)	<0.001
	Endotracheal tube placement during event	78.5% (n=102)	21.5% (n=28)	
	No endotracheal tube usage	94.6% (n=70)	5.4% (n=4)	
	Reintubated	89.5% (n=17)	10.5% (n=2)	

## Discussion

Cardiac arrest is characterized by a sudden loss of breathing and heart function. If not treated immediately, poor blood supply to the brain and other organs can result in a person losing consciousness or even passing away [1]. This study reviewed 351 medical records of patients who underwent CPR at the KSMC emergency department. This group was comprised of 24.8% females and 75.2% males, with a median age of 56.6 years (±14.7 SD). It showed that 30.2% of the patients achieved ROSC, and 11.39% were discharged alive. Our study results showed a better CPR outcomes rate compared to a study done in Malaysia, where the CPR outcomes rate was 30.2% and 9.5% for ROSC and STD, respectively [9]. Another study done in Iran showed that of 551 in-hospital CPRs, only 141 (25.58%) were successful, which is lower than our study [11]. Our CPR outcome rate was slightly lower in comparison to the in-hospital CPR outcomes in a study conducted in Pakistan [12].

Some studies have shown how advancing age is associated with a reduced likelihood of survival to hospital discharge, whereas others claim that there is no association between age and the outcome of CPR [13,14]. The mean age in all patients, ROSC, and STD groups was 56.6, 52.17, and 48.3 years, respectively, revealing that there is a significant association between younger age and better outcomes post-CPR. Counseling families during and after CPR may be aided by using age as an independent prognostic factor for predicted outcomes.

Surprisingly, there was no association between the cardiac rhythm and ROSC/STD; this could be explained by the fact that the shockable rhythms in this study were 6.6% compared to other studies that were conducted in Pakistan and Norway that included 25% and 27% shockable rhythms, respectively [12,15]. Adults who experience cardiac arrest often start with a VF or VT rhythm, which eventually degenerates into asystole [16,17]. However, that was not the case in our study. The same results were observed in a study done in Saudi Arabia, where shockable rhythms were 3.9% [18].

The largest number of resuscitated patients in this study (75.2%) were male. Contrary to a study done in Iran [19], the results showed no significant relationship between the outcome of CPR and gender. In addition, our study showed that a delay in epinephrine administration does not affect the outcome of CPR. Many studies have tried to find out the role of epinephrine administration and CPR; however, the utility of epinephrine administration in patients with cardiac arrest remains controversial [20-25].

In our study, we demonstrated a significant association between endotracheal intubation and favorable CPR outcomes, which is in line with a study done in China [26]. However, in a study done in Saudi Arabia, they found endotracheal intubation during CPR in the ED was associated with worse ROSC and survival to hospital discharge at 28 days [27].

Although this study provides valuable insights into the predictors of favorable CPR outcomes in the emergency department, there are some limitations that should be considered. First, this study was a retrospective analysis, and as such, the data collected was reliant on medical records. Therefore, this could potentially lead to inaccuracies in the data, as not all the data points may have been accurately recorded. Additionally, this study was conducted in only one center, which may limit the generalizability of the results. Several patients lacked post-hospitalization patient data, making it impossible to study the relationship between CPR and survival and longer-term benefits. Finally, the sample size of this study was relatively small, with only 351 patients included. This may limit the statistical power of the analysis and lead to potential biases in the results.

## Conclusions

This was a retrospective study carried out at KSMC that focuses on identifying the outcome of CPR in cardiac arrest patients presented to the emergency department between January 2017 and January 2020 and identifying predictors of these outcomes. Comparing the study's findings in relation to those of similar studies, it shows a CPR outcome rate within the similar studies' range. It also highlights that CPR outcomes are associated with CPR duration not exceeding 30 minutes, younger age, and endotracheal intubation. Multi-center prospective studies should be conducted in the future, enabling standardized recording of events and prognostic factors, enabling more precise knowledge of the factors that predict CPR outcomes, and increasing the generalizability of the results.
